# An Approach towards Motion-Tolerant PPG-Based Algorithm for Real-Time Heart Rate Monitoring of Moving Pigs

**DOI:** 10.3390/s20154251

**Published:** 2020-07-30

**Authors:** Ali Youssef, Alberto Peña Fernández, Laura Wassermann, Svenja Biernot, Eva-Maria Wittauer, André Bleich, Joerg Hartung, Daniel Berckmans, Tomas Norton

**Affiliations:** 1 Faculty of Bioscience Engineering, Katholieke Universiteit Leuven (KU LEUVEN), Kasteelpark Arenberg 30, 3001 Heverlee/Leuven, Belgium; ali.youssef@kuleuven.be (A.Y.); alberto.penafernandez@kuleuven.be (A.P.F.); daniel.berckmans@kuleuven.be (D.B.); 2 Institute for Laboratory Animal Science and Central Animal Facility, Hannover Medical School, Carl-Neuberg-Str. 1, 30625 Hannover, Germany; pferde.und.voegel@web.de (L.W.); s.biernot@web.de (S.B.); Wittauer.Eva-Maria@outlook.de (E.-M.W.); Bleich.Andre@mh-hannover.de (A.B.); 3 University of Veterinary Medicine Hannover, Foundation, 30559 Hannover, Germany; Joerg.Hartung@tiho-hannover.de

**Keywords:** pig′s heart rate, photoplethysmography (PPG), continuous wavelet transform (CWT), motion artefacts

## Abstract

Animal welfare remains a very important issue in the livestock sector, but monitoring animal welfare in an objective and continuous way remains a serious challenge. Monitoring animal welfare, based upon physiological measurements instead of the audio–visual scoring of behaviour, would be a step forward. One of the obvious physiological signals related to welfare and stress is heart rate. The objective of this research was to measure heart rate (beat per minutes) in pigs with technology that soon will be affordable. Affordable heart rate monitoring is done today at large scale on humans using the Photo Plethysmography (PPG) technology. We used PPG sensors on a pig′s body to test whether it allows the retrieval of a reliable heart rate signal. A continuous wavelet transform (CWT)-based algorithm is developed to decouple the cardiac pulse waves from the pig. Three different wavelets, namely second, fourth and sixth order Derivative of Gaussian (DOG), are tested. We show the results of the developed PPG-based algorithm, against electrocardiograms (ECG) as a reference measure for heart rate, and this for an anaesthetised versus a non-anaesthetised animal. We tested three different anatomical body positions (ear, leg and tail) and give results for each body position of the sensor. In summary, it can be concluded that the agreement between the PPG-based heart rate technique and the reference sensor is between 91% and 95%. In this paper, we showed the potential of using the PPG-based technology to assess the pig′s heart rate.

## 1. Introduction

Animal welfare remains a very important issue in the livestock sector, but monitoring animal welfare in an objective and continuous way remains a serious challenge. The continuous monitoring of autonomic nervous system activity in farm animals is now gaining considerable attention worldwide. The vagal component of the autonomic nervous system in the farm animals plays a key role in regulating heart rate (HR) in response to external and internal stressors [1,2,3]. Variables derived from cardiac activity are becoming increasingly important in research into animal health and wellbeing. In general, the variable heart rate (HR) can be used to indicate disease and physiological and psychological stress, and to show the individual characteristics of animals such as temperament and coping strategies. Additionally, for homoeothermic living organisms in general, the heart rate (HR) is a crucial variable (actuator) in controlling the metabolic energy production of the body. This includes the basal metabolism, the thermal component for controlling internal body temperature against external perturbations (thermoregulation) [4,5,6], the physical or mechanical component and the mental component, which is a key component in transferring feed energy efficiently into production and to preventing depression of the immune system due to stress [7]. The less efficiently metabolic energy is used in the body, the more feed energy will be wasted in manure, emissions, stress systems, etc. As such, animal HR is considered an important variable in health and welfare studies of farm animals [7]. However, it remains a challenge to monitor HR accurately and continuously via a reliable, affordable sensor on the animal, or with a remote sensing technique.

In commercially reared pigs, psychological stress may develop as their natural behaviour is usually strongly restricted. For example, sows during lactation are usually restricted from free movement and not provided with the required materials to build their nest. Such behavioural restriction may be a source of stress. Other stressors, like feed restriction in pregnant females, varying ambient temperatures and social restriction, are common in reproduction pigs [8]. Additionally, Von Borell et al. [8] showed that heart rate is a suitable indicator of stress in pigs. Hence, the real-time monitoring of the pig′s heart rate can provide vital information on how to maintain optimal conditions for production and animal welfare.

Currently, the heart rate of pigs can be monitored in two different ways: with implantable transmitters or with externally mounted non-invasive transmitters. The first method has the disadvantage that the implantation of the transmitters needs to be done under complete anaesthesia. This means that the pigs need a couple of days of recovery after the procedure. Furthermore, complications during the procedure can emerge. For the second method, a portable monitoring system that can detect and store electrocardiograms (ECG) for later detection of inter-beats intervals is commonly used. The equipment can consist of three coated electrodes mounted around the thorax. The disadvantage is that the mounting technique is practically not feasible under field conditions, due to the expected interactions between the animals. Additionally, and due to such interactions, the acquired signal can be disrupted by the movement of the electrode belt either by other pigs or the pig itself [8]. ECG measurements in pigs, as an excellent model for human cardiovascular diseases, are used experimentally in many research works due to the similarities in heart characteristics of humans and pigs [8].

Photoplethysmography (PPG) is a low-cost optical technique that can be used to detect heart beat rate based on changes in the volumetric blood flow. Currently, there is a growing interest in the real-time, wearable and ambulatory monitoring of human vital signs using PPG sensors [9]. Similarly, the same interest is valid for animal health monitoring as well. The use of the PPG-based technique in animal applications is so far limited, except for in some experimental studies (e.g., [10]). In a recent review study [7], Nie et al. showed the potential of transferring the PPG-based technique, which is successfully applied in human beings, to livestock. One important issue pointed out by Nie et al. [7] is whether the PPG theory, based on skin blood perfusion, is applicable for animals, which is an issue that depends mainly on the similarities of skin between humans and animals. Many studies, which are summarised in [7], documented several anatomical and physiological similarities between pigs and humans. Based on these anatomical and physiological similarities of the porcine skin to human skin, Nie et al. concluded in their study [7] that the PPG theory has potential applicability in heart rate assessment for pigs. To develop such PPG-based system for pigs, several factors that affect the reliability of the PPG signal should be considered, such as motion artefacts removal and measurement site on the body. Motion and noise artefacts are obstacles to collecting high quality signals that can be used for the clinical diagnosis of certain diseases and health conditions. Baseline drifting and motion artefacts are the limitation obstacles to using the PPG for diagnosis, as the noise can limit the practical implementation and reliability of real-time monitoring applications [9]. Motion artefacts in signals are considered as manifestations of the relationship between motion and noise. Voluntary and involuntary movements of the interface between the sensor and tissue [11] are the main cause of motion artefacts. Furthermore, anatomical and morphological regional variations in the skin constitute another reason for the varying PPG signal quality. Many studies have been done to improve the PPG physical sensing components so as to decrease motion artefacts, yet more analysis is needed to determine which sensor location is the best for monitoring heart rates in animals. Recently, many studies (e.g., [9,12,13]) have focused on determining the clinical reliability of the PPG measurements and the optimal signal quality index (SQI) in assessing the PPG signals, especially for mobile health and real-time applications.

The present paper presents a proof-of-concept study, with the main goal of testing the possibility of using the PPG-based technique to assess a pig′s heart rate and to determine the optimal location on the pig′s body that gives the best PPG signal quality. Additionally, we also sought to develop a real-time monitoring algorithm to extract a pig′s heart rate from a PPG signal, and to minimise the effects of motion artefacts.

## 2. Materials and Methods

### 2.1. Experimental Setup and Measurements

Over the course of this study, all measurements were conducted on a female *Göttinger Minipig* (test pig) under both anaesthetised and non-anaesthetised conditions. The test pig was born on 28.04.2017, with a 0.99 m back length (nose to tail) and weighing 30.2 kg on the day of experiments. The experiments were conducted in the Institute for Laboratory Animal Science, Hannover Medical School, Hannover, Germany. The measurements were obtained as part of a medical experiment to investigate and optimise liver cell transplantation. The original study and all measurements were ethically approved by “*Niedersächsisches Landesamt für Verbraucherschutz und Lebensmittelsicherheit*” (LAVES) (Germany; 33.12-42502-04-16/2374). Due to predefined planning in the ethical committee document, only one test pig was assigned for this study, which is considered enough for a proof-of-concept and pilot study.

Test on anaesthetised pig

**Figure 1:**
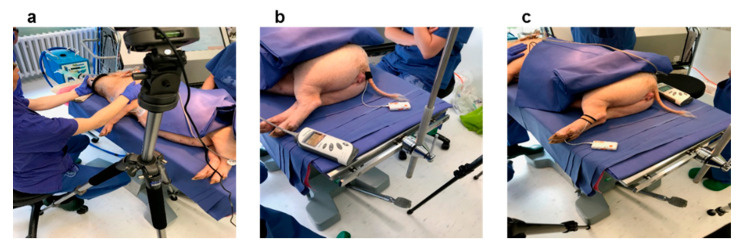
The test pig under anaesthetisation with PPG sensor placed on the left ear (**a**), on the tail (**b**) and on the left back leg(**c**).

During this part of the experiment, the pig was anaesthetised to measure the baseline maximum liver function capacity prior to liver resection (LiMAx measurement). The test pig underwent a total period of anaesthesia of one hour. These baseline measurements are always taken 5 days before the operation. To anaesthetise the pig, Zoletil (Tiletamin and Zolazepam, each mg.kg^−1^ i.m.) and Atropine (0.04–0.08 mg.kg^−1^ i.m.) were used. The timing of this part of the experiment was 60 min after the awakening procedures of the anaesthetised pig took place. This time course of 60 min was divided into three time slots, 20 min each. For each time slot, the PPG sensor probe was placed on three different anatomical sight/locations of the test pig (Figure 1), namely ear, upper tail and left back leg (below the knee). These locations of the pig′s body were chosen because of their higher cutaneous perfusion, and being a place where body fat is low yet still suitable to place the sensor probe in practice.

Test on non-anaesthetised pig (moving pig)

**Figure 2:**
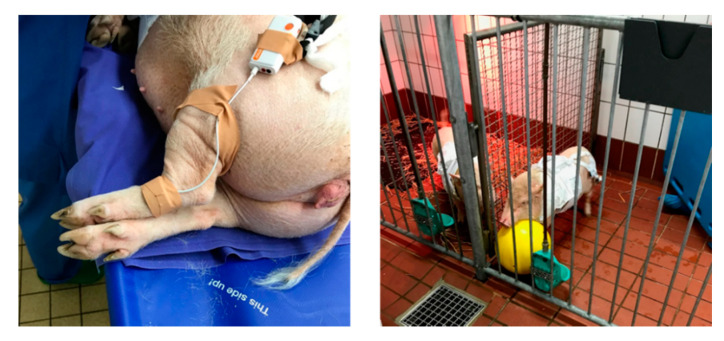
The PPG sensor is placed on the left back leg (below the knee) of the non-anaesthetised pig (left photo) and then the animal is allowed to move freely in a pen (right photo).

After about one hour (65 min) of applying the awakening procedure on the test pig, the PPG probe was placed on the left back leg (below the knee; Figure 2). Due to some practical difficulties we could not keeping a good contact between the PPG sensor and the skin of the pig′s ear and tail because of the pig′s movement. Then the test pig was allowed to move freely inside a test pen (Figure 2, right photo) with about 4.5 m^2^ total surface area. The duration of the test was 60 min in total. During the whole period of the test, continuous medical and ethological observations were performed by the trained staff (Laboratory Animal Science, Hannover Medical School, Hannover). Throughout the test period, it was noticed that the pig was freely moving and playing, with no events of laying on the floor, drowsiness or stress.

#### 2.1.1. Measurements and Sensors

##### PPG Signal

**Figure 3:**
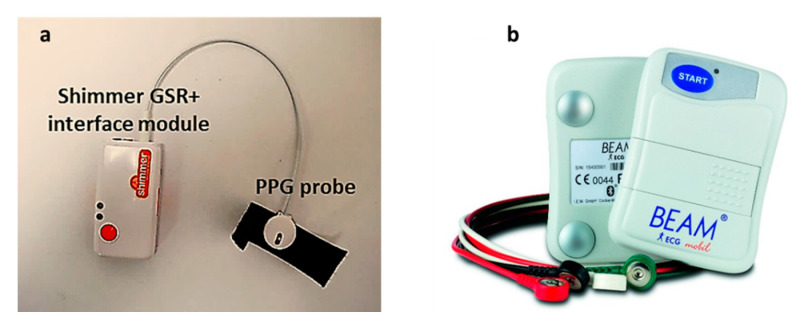
(**a**) the shimmer Optical Pulse sensing probe (PPG sensor) and data-logger, (**b**) and the ECG recorder, BEAM^®^, used to record ECG signals from the pig as a gold standard for heart rate.

A Shimmer Optical Pulse sensing probe (PPG sensor) together with Shimmer GSR+ module (Figure 3a) were used to collect the PPG signal from the pig. The PPG signal is acquired at a sampling rate of 128 Hz.

##### Gold Standard (ECG Signal)

As a gold standard for heart rate measurements, continuous ECG measurements were performed. The ECG measurements were performed using a portable ECG recorder: BEAM^®^ ECG 3-channels (Figure 3b) Loop/Event recorder (IEM GmbH; Stolberg, Germany). It is an on-body portable ECG recorder, with three electrodes to stick on to the skin. The recorded ECG signal is automatically transferred from the BEAM^®^ via Bluetooth to a smartphone, and is forwarded from there to a secure database. The BEAM^®^ recorded the ECG data every 0.6 s.

### 2.2. Signal Processing and Heart Rate Extraction

**Figure 4:**
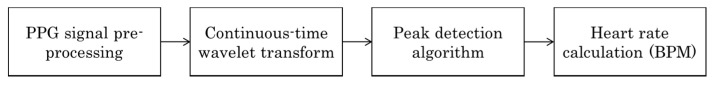
Block diagram showing the main processing steps to extract pig′s heart rate from PPG signal.

Figure 4 shows the main steps of (pre-) processing methodologies to extract the pig′s heart rate from the acquired PPG signals. The proposed algorithm consists of four main processing blocks. Each block is explained in detail in the following sections.

#### 2.2.1. Pre-Processing of PPG Signals

The PPG signals are mostly affected by different sources of noise, such as surrounding lights and motion artefacts (in the non-anaesthetised case). Therefore, firstly, the signals are normalised to zero mean and unit variance [14]. Then, the normalised signals are filtered using a second order zero-phase Butterworth high pass filter (cut-off frequency of 0.5 Hz) and a first order zero-phase Butterworth low pass filter (cut-off frequency of 6 Hz). These cut-off frequencies were chosen based on the expected physiological heart rate range. The Butterworth filter provides a maximally flat passband together with the zero-phase implementation which is preserving the pig′s cardiac wave. The second derivative of the PPG signal, also called the acceleration plethysmogram (APG), shows more defined peaks than those of the PPG signal, and can therefore be used as a more accurate detector of heart rate [15].

#### 2.2.2. Wavelet Analysis and Cardiogenic Signal Reconstruction

In the medical and biomedical engineering fields, wavelet transform (WT) is often preferred over Fast Fourier Transform (FFT) in signal processing and the detection of cardiac waves. This is due to the fact that the physiological signals are naturally non-stationary, which makes WT a viable and powerful technique for biological and medical application. The wavelet transform is a suitable technique for analysing time series that contain nonstationary power at many different frequencies [16,17]. Using WT, the signals in the time domain are mapped onto the frequency domain in order to preserve both the time and frequency information. WT is a spectral estimation technique that functions by breaking a general function into an infinite series of wavelets [14].

##### Continuous Wavelet Transform Method

Generally, in the continuous wavelet transform (CWT) method, a specific wavelet centred around a given frequency is computed from the mother wavelet by scaling and shifting it. In this manner, the length of the wavelet contains the same number of centre (also called peak) frequency cycles. For a scale parameter, s>0, and a position parameter, b, which defines a translation of the wavelet and indicates the time localisation, the CWT can be given by:

(1) $$C\left(s,b\right)=\int_{-\infty }^{+\infty }x\left(t\right)\frac{1}{\sqrt{s}}\psi^{*}\left(\frac{t-b}{s}\right)dt$$

The wavelet analysis is performed by convoluting a signal, x(t), with a certain mother wavelet, ψ(t). The $\psi^{*}\left(t\right)$ is the complex conjugate of the analysing mother wavelet. The term $\frac{1}{\sqrt{s}}$ is an energy normalised factor (the energy of the wavelet must be the same for different s values of the scale). As the scale, s, increases, the wavelet is compressed, its spectrum dilates, and the peak frequency shifts to a higher value. Conversely, when s decreases, the wavelet dilates, its spectrum is compressed and the peak frequency shifts to a lower value. In practice, the CWT is computed over a discrete value of the scale ‘s′ in the range of continuous values. Thus, to approximate the continuous wavelet transform, the Equation (1) should be calculated N times for each scale, where N is the number of points in the discrete signal x(t) [17]. In general, the classic CWT transform is time-consuming and it requires too much computing power to be applied in real-time. Hence, more efficient algorithms have been developed to reduce the required computational power and the time of CWT calculation (e.g., [18,19,20]).

In this paper, the CWT is calculated using fast Fourier transform (CWFT) [20], which allows us to compute the N convolutions simultaneously, which is more suitable for real-time applications. The CWFT algorithm implements the following steps:

- Compute the discrete Fourier transform (DFT) of the analysed signal x(n), which includes N samples, using Fast Fourier Transform (FFT) as follows:
  (2) $$\hat{x}\left(k\right)=\sum_{n=0}^{N-1}x\left(n\right)e^{-i\frac{2\pi }{N}nk},\;k=0,1,2\cdots N-1$$
  where k is an index of frequency.
- Obtain the DFT ($\hat{\psi }$) of the analysing wavelet (ψ) at the appropriate angular frequencies as follows:
  (3) $$\hat{\psi }\left(k\right)=\sum_{n=0}^{N-1}\psi \left(n\right)e^{-i\frac{2\pi }{N}nk},\;k=0,1,2\cdots N-1$$
- Obtain the DFT of the analysing wavelet ψ(n) at different scales.

To maintain a unit of energy for each scale s, the wavelet function is normalised by the following formula:

(4) $$\hat{\psi }\left(s\omega_{k}\right)=\sqrt{\frac{2\pi s}{\Delta t}}\hat{\psi }\left(s\omega_{k}\right),$$

where $\Delta t=1/f_{s}$ is the sampling period, with $f_{s}$ as the sampling frequency and $\omega_{k}=\frac{2\pi k}{N\Delta t}$.

Compute the product of the signal DFT and the wavelet DFT over all the defined scales. Invert the DFT to obtain the CWT coefficients as follows:

(5) $$W_{s}\left(b\right)=\frac{1}{N}\sqrt{\frac{2\pi s}{\Delta t}}\overset{N-1}{\underset{k=0}{\sum }}\hat{x}\left(\frac{2\pi }{N\Delta t}k\right){\hat{\psi }}^{*}\left(s\frac{2\pi }{N\Delta t}k\right)e^{i\frac{2\pi }{N}kb}$$

During the initial phase of processing, the aforementioned CWTFT algorithm is applied to decouple the cardiogenic pulsatile signals from the measured PPG signals based on various wavelets, namely non-analytical Mortlet, *m*-th order Derivative of Gaussian (DOG) Bump and Paul. Based on the initial signal processing of the acquired PPG signals from the pig, the *m*-th order Derivative of Gaussian (DOG) wavelets were chosen for the presented work because, in general, the obtained scalograms (scales “s” vs. positions “b”) using these wavelets showed clear frequency contents within the expected pulse rate ranges of the different strains of pigs [7].

**Figure 5:**
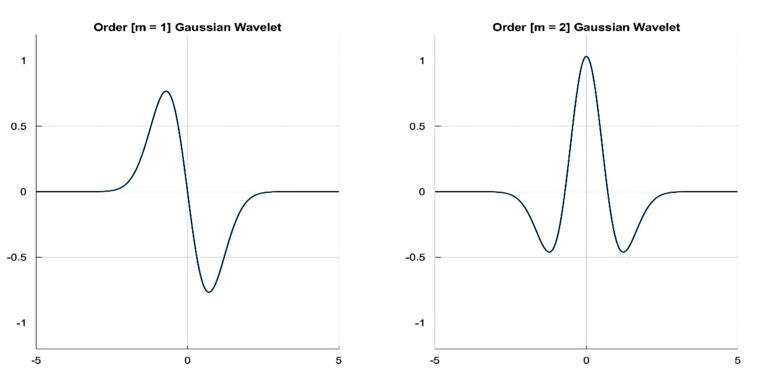
First and second order derivatives of Gaussian wavelets as examples of odd and even Gaussian wavelets.

The Gaussian function is perfectly local in the time and frequency domains, and a derivative of any order (m) of the Gaussian function may be a wavelet transform (WT) since it is indefinitely derivable. A typical cardiac pulse event in the PPG signal consists of two modulus maxima with different signs of $W_{s}\left(s,b\right)$ (i.e., maxima and minima) [21]. Sahambi et al. [21] used a first order (m=1) odd function (Figure 5) to detect the QRS complex in the ECG signal. However, in our case here, the minima of the cardiac event in the measured PPG is found to be distorted in most of the cases. Therefore, only the maxima are used to compute the pig′s heart rate (HR) from the measured PPG. Hence, in this paper, an even order (with order m={2z : z ∈ ℤ}) derivative Gaussian (DOG) wavelet is used to decouple the cardiogenic (pulsatile) PPG signal using the CWTFT algorithm. 

Three orders of the DOG wavelet, namely, m = 2, 4 and 6, were tested to investigate the most suitable one for computing the HR from the acquired PPG signals. The processing and analysis of the signals is done using custom script written in MATLAB (The Math Works, Inc., Natick, Massachusetts, USA) based on the *Signal Processing* and *Wavelet Analyser* toolboxes.

#### 2.2.3. Signal Quality Indices (SQIs)

The perfusion index ($P_{SQI}$) is presented as the gold standard in many research works (e.g., [9,22,23,24]) for assessing PPG signal quality. In their work [9], Elgendi et al. used a statistical approach to find out the optimal SQI for the quality assessment of PPG samples out of eight different SQIs, of which the “skewness” index ($S_{SQI}$) was said to perform best. Additionally, Krishnan et al. [25] found out that the skewness can be associated with corrupted PPG signals. In many science and engineering applications, signal to noise ratio ($NS_{SQI}$) is a standard measure that compares the level of a desired signal to the level of background noise, which can be a good indicator of PPG signal quality. In this paper, we used the three aforementioned signal quality indices, namely, perfusion index ($P_{SQI}$), skewness index ($S_{SQI}$) and signal to noise ratio index ($SN_{SQI}$) to assess the quality of the PPG signal acquired from different body locations of the pig. The used SQIs are defined as follows:

- *Perfusion index* ($P_{SQI}$) is defined as the ratio of the pulsatile signal component to the non-pulsatile or static blood flow in the peripheral tissue. In other words, it is the difference in the amount of light absorbed by the pulse when light is transmitted through the finger [9], which can be defined as follows:
  (6) $$P_{SQI}=\left[\left(X_{max}-X_{min}\right)/\left|\bar{x}\right|\right]\times 100$$
  where $\bar{x}$ is the statistical mean of the x signal (raw PPG signal), and X is the filtered PPG signal.
- *Skewness index* ($S_{SQI}$) is a measure of the symmetry/asymmetry of a probability distribution of the signal about its mean, which is defined as:
  (7) SSQI=1N∑n=1N[xn−μ^x/σ]3
  where ${\hat{\mu }}_{x}$ and σ are the empirical estimate of the mean and standard deviation of $x_{i}$, respectively, and N is the number of samples in the PPG signal [9].
- *Signal to Noise ratio* ($SN_{SQI}$) compares the level of a desired signal (pulsatile cardiogenic signal) to the level of background noise [9], and is given by
  (8) $$SN_{SQI}=\frac{\sigma^{2}{}_{x}}{\sigma^{2}{}_{\mathrm{noise}}}$$
  where $\sigma_{x}$ is the standard deviation of the absolute value of the PPG signal (*x*), and $\sigma_{\mathrm{noise}}$ is the standard deviation of the *noise*.

#### 2.2.4. Peak Detection and Heart Rate Calculation

In general, the heartbeat could be estimated by calculating the time between the peak intervals in the PPG signal. The peak is detected by calculating the local maxima of the decoupled cardiac pulse signal X(n) within a predefined interval (window) I, so that by finding $n_{o}\in I$ we fulfil that

$$X\left(n_{o}\right)\geq X\left(n\right),\;\forall n\in I$$

The algorithm then repeats the procedure of the tallest peak and iterate until it runs out of considerable peaks.

**Figure 6:**
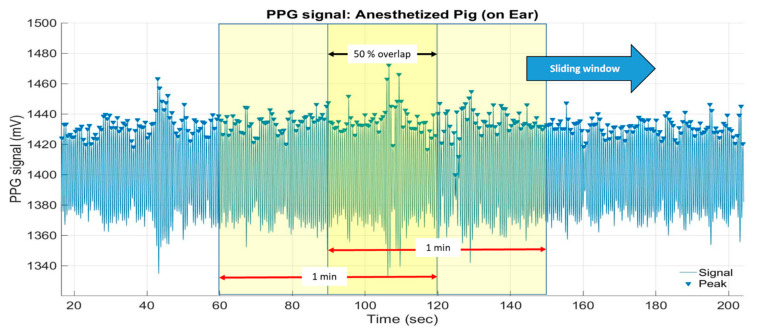
One-minute sliding window with 50% (30 s) overlap to calculate the heart rate (bpm) based on the number of detected peaks per time window.

The heart rate in bpm is calculated based on the number of detected peaks within a sliding time window or an epoch of one minute, with 30 s (50%) overlap (Figure 6).

## 3. Results and Discussion

### 3.1. Decoupling of the Pulse Wave in the Anaesthetised Pig

All the measured PPG signals obtained from the ear, leg and the tail of the anaesthetised pig are divided into segments of 60 s (i.e., 2560 data samples) to be processed individually. After the preprocessing step (see Section 2.2.1), the continuous wavelet transform of each segment is computed using the CWTFT algorithm. The most tricky steps in decoupling the cardiogenic pulse wave using CWT are, first, choosing the optimal suitable set of scales (s), and second, choosing the suitable mother wavelet.

#### 3.1.1. Scales Selection

**Figure 7:**
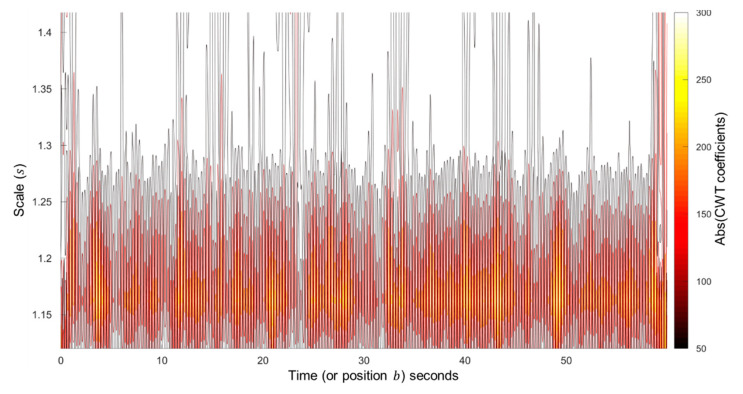
The scalogram of the chosen set of scales ($s=2^{a}$) shows the scales dominated by the energy (high absolute CWT coefficients) from the cardiogenic pulse signal, calculated using the fourth order DOG wavelet.

The scale (s) parameter is the set of real powers of 2, i.e., $s=2^{a}\;where\;\left\{a\in \mathbb{Z}\right\}$. The suitable set of scales should contain most of the energy of the cardiogenic pulse wave. We found that the energy of the cardiogenic pulse signals, in all the PPG segments, is dominated by the scales between $2^{0.16}$ and $2^{0.50}$. Therefore, a set of five scales (s), namely $\left[2^{0.16}\;2^{0.21}\;2^{0.28}\;2^{0.38}\;2^{0.50}\right]$, is chosen for the wavelet calculation. An example scalogram showing the distribution of the calculated CWT coefficients, using the fourth order DOG wavelet, over the chosen set of scales is depicted in Figure 7.

#### 3.1.2. Mother Wavelet Selection

As explained earlier, even order (with order m={2z : z ∈ ℤ}) derivative Gaussian (DOG) wavelets are chosen to calculate the CWT of the PPG signals. In particular, three orders of the DOG wavelet, namely, m = 2, 4 and 6, are tested. In the Fourier domain, the *m*-th order derivatives of Gaussian wavelets, DOG, are defined by:

(9) ψ^(sω)=1Γ(m+1s)(jsω)me−(sω)22,

where Г denotes the gamma function.

**Figure 8:**
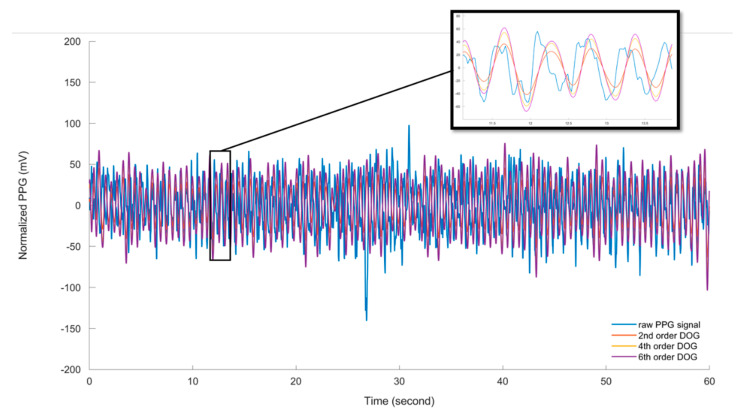
The decoupled cardiac pulse signals using the CWTFT algorithm based on three different orders (m = 2, 4, 6) of DOG wavelet in comparison to one segment of the original raw PPG signal obtained from the ear of the anaesthetised pig.

For each PPG data segment obtained from the ear, leg and the tail of the anaesthetised pig, the CWT are computed using the CWTFT algorithm, using DOG wavelets with the three selected orders. Figure 8 shows an example of the decoupled cardiac pulse waves using the CWTFT algorithm and the three DOG wavelets (second, fourth and sixth order DOG wavelets) from one segment of PPG signal obtained from the ear of the anaesthetised pig. Based on the decoupled cardiac pulse waves, resulted from the CWTFT algorithm, the pig′s heart rate (bpm) is computed using the peak detection algorithm. The estimated heart rate from the PPG signal is compared with the ground-truth heart rate (reference), which is calculated from the gold standard ECG signal. The performance of the developed algorithm is then evaluated in terms of the quality of the pulse rate estimation, which is assessed using the Mean Absolute Error (MAR) and the Root Mean Square Error (RMSE), which are given as follows:

(10) $$MAE=\frac{1}{N}\sum_{n=1}^{N}\left|HR_{PPG}\left(n\right)-HR_{ECG}\left(n\right)\right|,$$

(11) $$RMSE\;=\sqrt{\frac{1}{N}\overset{N}{\underset{n=1}{\sum }}{\left(HR_{PPG}\left(n\right)-HR_{ECG}\left(n\right)\right)}^{2}}$$

where N is the total number of data points, and $HR_{PPG}\left(n\right)$ and $HR_{ECG}\left(n\right)$ are the estimated heart rate from the PPG signal and that calculated from the ECG, respectively, at time instant n. RMSE is more sensitive to large estimation errors than MAE, so a small number of large errors results in high RMSE and low MAE. Table 1 shows a comparison of the estimated heart rate (HR) using three DOG wavelets (second, fourth and sixth order DOG wavelets) based on the MAR and RMSE values calculated for each PPG segment obtained from the anaesthetised pig. The results show that there are significant (*p* < 0.05) differences in both the MAR and RMES values for all the estimated HR values using the second order DOG wavelet and those estimated using the fourth and sixth order DOG wavelets. The results did not show a significant difference between the fourth and sixth order DOG wavelets in either the MAR or RMES values. As such, the fourth order DOG wavelet is suggested to decouple the cardiac pulse signals from the measured PPG using the CWTFT algorithm.

#### 3.1.3. Assessment of PPG Signal Quality

To assess the signal quality of each segment of the measured PPG signals obtained from the ear, leg and the tail of the anaesthetised pig, the cardiac pulse waves were decoupled using the CWTFT algorithm based on the fourth order DOG wavelet. Then, specific SQIs, namely perfusion index ($P_{SQI}$), skewness index ($S_{SQI}$) and signal to noise ratio index ($SN_{SQI}$), were computed for each segment and compared. Table 2 shows the three calculated SQI values of the PPG signals obtained from different pig body positions (ear, leg and tail) under both anaesthesia and no-anaesthesia conditions.

**Figure 9:**
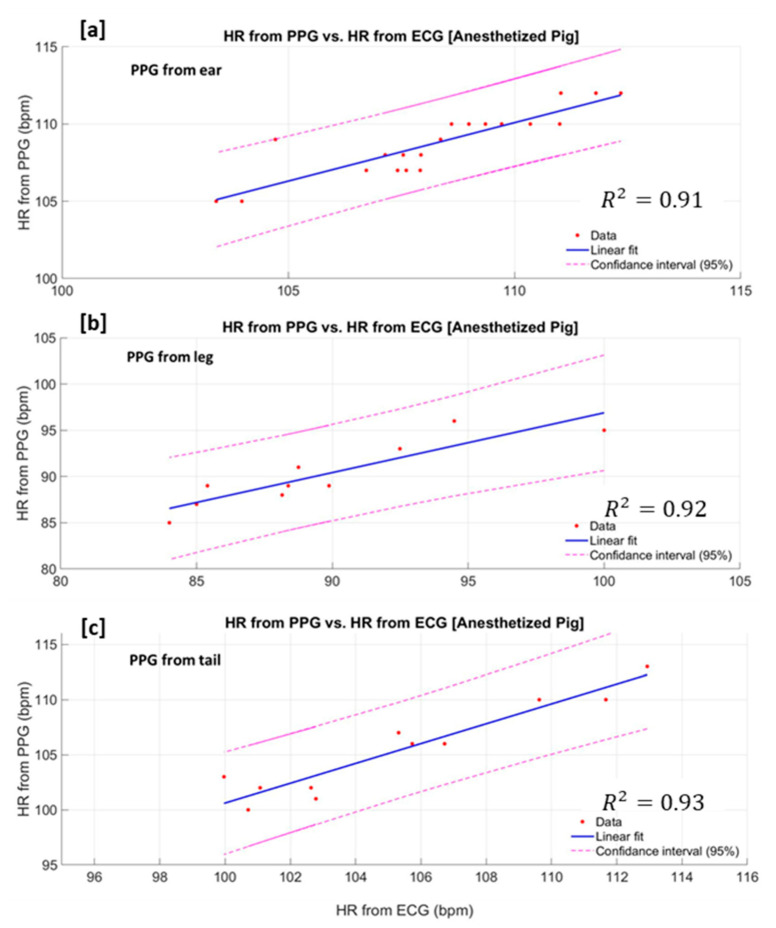
Estimated heart rate from PPG signal vs. heart rate from the gold standard (ECG) measured from the anaesthetised pig′s ear (**a**), the leg (**b**) and the tail(**c**).

The overall accuracy of the heart rate (HR) estimation algorithm is calculated for the PPG signal obtained from the ear, leg and the tail of the anaesthetised pig (Figure 9). The developed HR estimation algorithm is able to detect the heart rate from the pig′s ear, leg and tail with overall accuracies of 91%, 92.4% and 93.2%, respectively.

### 3.2. Heart Rate Estimation Based on Measured PPG From the Non-Anaesthetised (Moving) Pig

**Figure 10:**
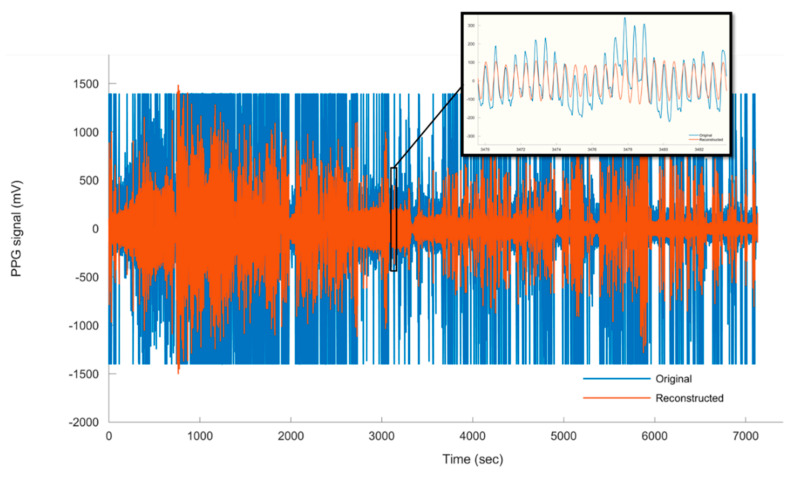
The raw PPG signal vs. the decoupled (reconstructed) cardiac pulse waves obtained from moving pig.

Due to the practical difficulty, we could not keep a good contact between the pig′s ear and the PPG sensor during the pig′s motion. Furthermore, to avoid the problem of tail biting, one of the largest animal welfare problems in modern pig production [26,27], attaching the PPG sensor to the pig′s tail is avoided. Hence, for the non-anaesthetised (moving) pig, PPG measurements are only obtained from the pig′s leg. The cardiac pulse waves from the moving pig are decoupled from the PPG signal using the CWTFT algorithm based on the fourth order DOG and the selected scales. The raw PPG signal showed more baseline wander and noises, which can be attributed to motion artefacts. However, the developed algorithm is successfully able to decouple the cardiac pulse waves (Figure 10). It is observed that most of the energy of the motion artefacts and baseline drifts increases for scales $s>2^{0.55}$, corresponding to frequencies <0.78 Hz.

**Figure 11:**
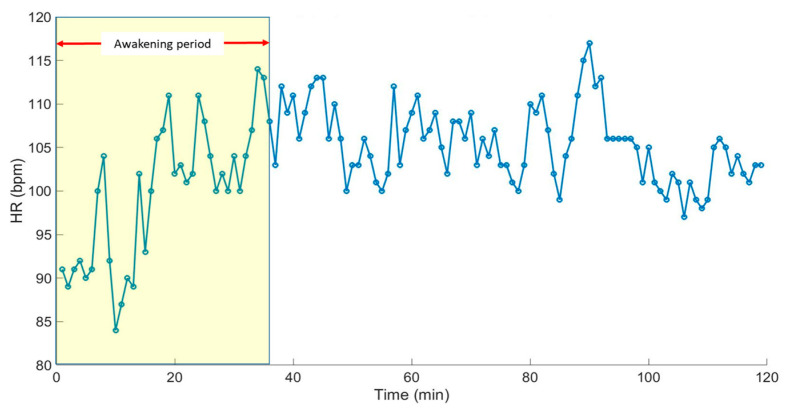
The estimated HR of the non-anaesthetised (moving) pig using the developed CWTFT-based algorithm along the whole measurement period, including the awakening period.

The heart rate (HR) of the moving pig is estimated using the peak detecting algorithm based on the decoupled cardiac pulse waves. Figure 11 shows the estimated HR based on the CWTFT algorithm for the whole measurement period, and highlights the evolution of the heart rate through the awakening period after the anaesthetised period. Then, the estimated heart rate from the PPG signal is compared with the ground-truth heart rate (reference) from the gold standard ECG signal.

The calculated Mean Absolute Error (MAE) and Root Mean Square Error (RMSE) of the estimated heart rate of the moving pig are 1.6(±0.8) bpm and 2.5 (±1.9) bpm, respectively. The algorithm successfully estimated the heart rate of the moving pig with an overall accuracy of 91%. This work illustrates the capacity of the developed PPG-based algorithm, as a proof-of-concept, and the possibility of using it to continuously monitor the pig′s heart rate in the field. Although the developed algorithm successfully estimated the heart rate, with high accuracy, of both an anaesthetised and a moving pig, the algorithm is still to be tested on a large population of pigs, considering the age, sex, weight and strain. However, the algorithm is a potential technique for real-time application in monitoring pig heart rates. In addition to pigs′ heart rates, the developed PPG-based algorithm can also be upgraded to calculate other vital health and welfare signs, such as animal heart rate variability (HRV) and respiration rate in the field. That said, most HRV studies in pigs are based on the pigs as models in biomedical research for human diseases, however some studies have considered its potential role in stress and welfare monitoring [8]. Hence, we are planning in future work to test the possibility of including the HRV and respiration rate in the proposed algorithm. In the last 10 years, the PPG-based sensors have been successfully applied in human-related applications that, because they are low cost and simple, can be developed with ultra-low power technology and be non-invasive. Therefore, the transfer of PPG-based techniques to livestock applications is promising. Nie et al. [7] pointed out one important consideration concerning whether the PPG theory based on skin blood perfusion is applicable for animals, which is an issue related to the similarities in skin characteristics between humans and animals. In the current work, we can conclude that the PPG theory is applicable to heart rate assessment for pigs. This can be attributed to the several documented [7] anatomical and physiological similarities between pig skin and human skin. However, it should be stated here that some technical and practical factors are to be considered, such as the contact pressure between the sensor and the animal skin, power consumption, weight/size, and last but not least, the cost. A detailed assessment of such challenging factors can be found in the intensive review work concerning the continuous heart rate monitoring of livestock by Nie et al. [7].

## 4. Conclusions

In this paper, a PPG sensor system is used to measure the heart rate (HR) of a *Göttinger Minipig* under anaesthetised and free-moving (non-anaesthetised) conditions. The PPG probe is placed on three different anatomical body positions, namely ear, leg and tail. The pulsatile cardiogenic signals are decoupled using a continuous wavelet transform (CWT). Three different wavelets, namely second, fourth and sixth order DOG, are tested. The 4th order DOG wavelet is found to be the most suitable mother wavelet for decoupling the cardiac pulse waves from both the anaesthetised and the free-moving pig. The peaks of the pulsatile cardiogenic signal are detected and pulse rates (bpm) are estimated from the PPG signals, using the developed algorithm. The results showed that the PPG signals obtained from the ear contained the highest SNR (3.85 ± 0.4 dB), while the PPG obtained from the tail contained the lowest SNR (3.51 ± 0.43 dB). The estimated HR of the free-moving pig (PPG probe placed on the leg) has shown RMSE and MAE values of 2.5 ± 0.4 bpm and 1.6 ± 0.8 bpm, respectively. In general, the developed CWTFT-based algorithm is able to decouple the pulsatile cardiogenic signals and estimate the pulse rate of the pigs from PPG signals obtained from the three different body positions, with accuracy levels between 91% and 95%. In this paper, we showed that the PPG theory is applicable to heart rate assessment for pigs. The developed algorithm represents a proof-of-concept for the real-time monitoring of a pig′s heart rate in the field using PPG-based technology. However, further investigations are needed to test the developed PPG-based algorithm on different and larger population, with consideration being paid to the animal age, sex, weight and strain.

## Figures and Tables

**Table 1:** The Mean Absolute Error (MAE) and Root Mean Square Error (RMSE) of the estimated heart rate (bpm) from all the PPG segments, in comparison to the reference pulse rate calculated from the ECG signals obtained from the anaesthetised pig. Comparing the estimated heart rate based on three orders (2, 4 and 6) of DOG wavelet.

	Wavelet	Ear	Leg	Tail
MAE (± std)	2nd order DOG	2.66 (±1.3) *	1.75 (±1.2) *	1.38 (±0.8) *
	4th order DOG	2.23 (±0.9)	1.53 (±0.8)	1.25 (±0.7)
	6th order DOG	2.20 (±1.1)	1.56 (±0.9)	1.32 (±0.8)
RMSE (± std)	2nd order DOG	3.50 (±1.6) *	2.27 (±1.2) *	1.45 (±0.9) *
	4th order DOG	3.10 (±1.4)	1.80 (±1.4)	1.39 (±0.6)
	6th order DOG	3.23 (±1.5)	2.11 (±1.6)	1.36 (±0.7)

* significant (*p* < 0.05).

**Table 2:** The average and standard deviation of the SQIs, perfusion index ($P_{SQI}$), skewness index ($S_{SQI}$) and signal to noise ratio index ($SN_{SQI}$) from all the PPG segments obtained from the ear, leg and the tail of the anaesthetised pig.

SQI	Ear	Leg	Tail
$P_{SQI}$	10 (±2.9)	8 (±2.2)	7 (±2.5)
$S_{SQI}$	0.06 (±0.09)	0.02 (±0.08)	0.02 (±0.09)
$SN_{SQI}$	3.85 (±0.40)	3.62 (±0.35)	3.51 (±0.43)
